# *N*-(4-Meth­oxy­phen­yl)-2,6-dimethyl-1,3-dioxan-4-amine

**DOI:** 10.1107/S1600536813023763

**Published:** 2013-08-31

**Authors:** Zeenat Fatima, Rambabu Gottimukkal, Bandapalli Palakshi Reddy, Vijayaparthasarathi Vijayakumar, Devadasan Velmurugan

**Affiliations:** a Centre of Advanced Study in Crystallography and Biophysics, University of Madras, Guindy Campus, Chennai 600 025, India; b Chemistry Department, GEBH, Sree Vidyanikethan Engineering College, A. Rangampet, Tirupati 517102, India; c Centre for Organic and Medicinal Chemistry, VIT University, Vellore 632 014, India

## Abstract

In the title compound, C_13_H_19_NO_3_, the dioxane ring adopts a chair conformation. Its mean plane is inclined to the 4-meth­oxy­phenyl ring by 70.34 (9)°. In the crystal, mol­ecules are linked by pairs of C—H⋯O hydrogen bonds, forming inversion dimers with an *R* ^2^_2_(16) ring motif. The dimers are linked *via* C—H⋯π inter­actions, forming two-dimensional networks lying parallel to the *ac* plane.

## Related literature

For biological properties of oxygen heterocycles, such as dioxane derivatives, see: Aubele *et al.* (2005 ▶); Marucci *et al.* (2005 ▶). For some applications, see: Wang *et al.* (1994 ▶, 1996*a* ▶,*b* ▶); Yuan *et al.* (2005 ▶). For related crystal structures, see: Chuprunov *et al.* (1981 ▶); Yuan *et al.* (2008 ▶). For graph-set motifs, see: Bernstein *et al.* (1995 ▶).
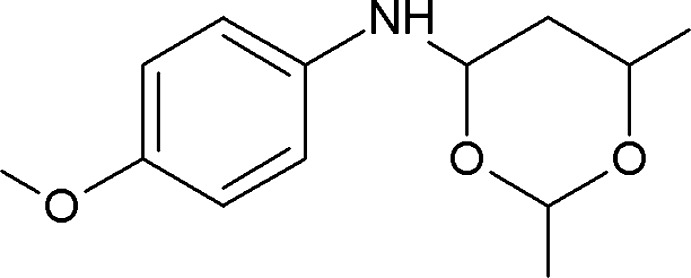


## Experimental

### 

#### Crystal data

C_13_H_19_NO_3_
*M* *_r_* = 237.29
Monoclinic, 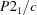

*a* = 9.6472 (6) Å
*b* = 13.8194 (8) Å
*c* = 10.5384 (6) Å
β = 114.355 (3)°
*V* = 1279.93 (13) Å^3^
*Z* = 4
Mo *K*α radiation
μ = 0.09 mm^−1^
*T* = 293 K
0.25 × 0.20 × 0.15 mm

#### Data collection

Bruker SMART APEXII area-detector diffractometer
Absorption correction: multi-scan (*SADABS*; Bruker, 2008 ▶) *T*_min_ = 0.667, *T*_max_ = 0.746
12259 measured reflections
3169 independent reflections
2133 reflections with *I* > 2σ(*I*)
*R*_int_ = 0.033

#### Refinement

*R*[*F* ^2^ > 2σ(*F* ^2^)] = 0.053
*wR*(*F* ^2^) = 0.162
*S* = 1.03
3169 reflections
158 parameters
H atoms treated by a mixture of independent and constrained refinement
Δρ_max_ = 0.26 e Å^−3^
Δρ_min_ = −0.21 e Å^−3^

### 

Data collection: *APEX2* (Bruker, 2008 ▶); cell refinement: *SAINT* (Bruker, 2008 ▶); data reduction: *SAINT*; program(s) used to solve structure: *SHELXS97* (Sheldrick, 2008 ▶); program(s) used to refine structure: *SHELXL97* (Sheldrick, 2008 ▶); molecular graphics: *ORTEP-3 for Windows* (Farrugia, 2012 ▶); software used to prepare material for publication: *SHELXL97* and *PLATON* (Spek, 2009 ▶).

## Supplementary Material

Crystal structure: contains datablock(s) global, I. DOI: 10.1107/S1600536813023763/su2641sup1.cif

Structure factors: contains datablock(s) I. DOI: 10.1107/S1600536813023763/su2641Isup2.hkl

Supplementary material file. DOI: 10.1107/S1600536813023763/su2641Isup3.cml

Additional supplementary materials:  crystallographic information; 3D view; checkCIF report

## Figures and Tables

**Table 1:** Hydrogen-bond geometry (Å, °) *Cg*1 is the centroid of ring C2-C7 ring.

*D*—H⋯*A*	*D*—H	H⋯*A*	*D*⋯*A*	*D*—H⋯*A*
C6—H6⋯O3^i^	0.93	2.51	3.407 (2)	162
C1—H1*B*⋯*Cg*1^ii^	0.96	2.77	3.700 (3)	163

Symmetry codes: (i) 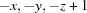; (ii) 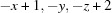.

**Fig. 1.**
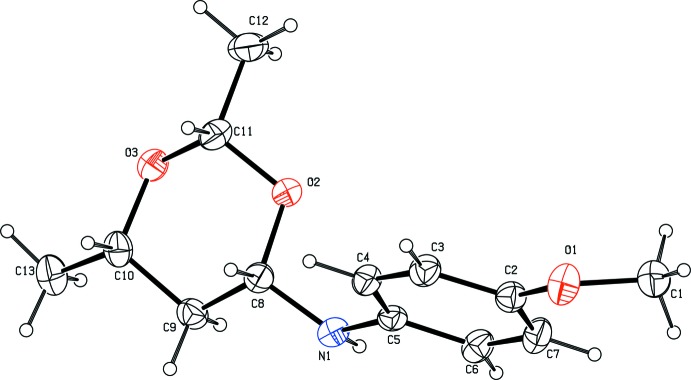
The molecular structure of the title molecule, with atom labelling. Displacement ellipsoids are drawn at the 30% probability level.

**Fig. 2.**
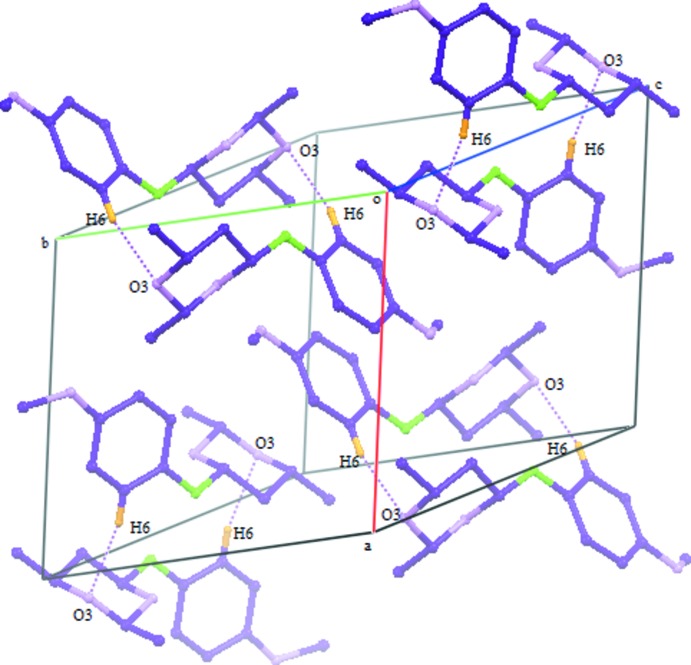
The crystal packing of the title compound viewed almost perpendicular to (011). Hydrogen bonds are shown as dashed lines (see Table 1 for details; H-atoms not involved in hydrogen bonding have been omitted for clarity).

## supplementary crystallographic information

### 1. Comment

Oxygen heterocycles play a vital role as basic building blocks in pharmaceutical preparations. Dioxane rings are frequently encountered in many bioactive molecules, such as cytotoxic agents (Aubele *et al.*, 2005) and antimuscarinic agents (Marucci *et al.*, 2005). This class of compounds also has useful insecticidal as well as anti-foaming properties (Yuan *et al.*, 2005; Wang *et al.*, 1994, 1996*a*,*b*). In view of the different applications of this class of compounds, we have synthesized the title derivative and report herein on its crystal structure.

In the title molecule, Fig. 1, the dioxane ring (O2/O3/C8—C11) adopts a *chair* conformation and its mean plane makes a dihedral angle of 70.34 (9)° with the benzene ring (C2—C7).

In the crystal, molecules are linked by pairs of C—H···O hydrogen bonds (Table 1 and Fig. 2) forming inversion dimers with an R^2^_2_(16) ring motif (Bernstein *et al.*, 1995). The dimers are linked via C-H···π interactions forming two-dimensional networks lying parallel to the ac plane (Table 1).

### 2. Experimental

To 4-anisidine (1 mmol), acetaldehyde (3 mmol) was added drop wise and stirred for about 4 h at 273 K. The progress of the reaction was monitored by TLC. The reaction mixture was then washed with petroleum ether. The residue was dissolved in diethylether and the solution left for the solvent to evaporate. The solid product obtained was recrystallized from diethylether giving block-like colourless crystals of the title compound.

### 3. Refinement

The NH H atom was located in a difference Fourier map and freely refined. The C-bound H atoms were placed in calculated positions refined as riding: C—H = 0.93 Å to 0.98 Å with *U*_iso_(H) = 1.5*U*_eq_(C-methyl) and = 1.2*U*_eq_(C) for other H atoms.

### Crystal data

**Table:**

C_13_H_19_NO_3_	*F*(000) = 512
*M**_r_* = 237.29	*D*_x_ = 1.231 Mg m^−^^3^
Monoclinic, *P*2_1_/*c*	Mo *K*α radiation, λ = 0.71073 Å
Hall symbol: -P 2ybc	Cell parameters from 3169 reflections
*a* = 9.6472 (6) Å	θ = 2.3–28.3°
*b* = 13.8194 (8) Å	µ = 0.09 mm^−^^1^
*c* = 10.5384 (6) Å	*T* = 293 K
β = 114.355 (3)°	Block, colourless
*V* = 1279.93 (13) Å^3^	0.25 × 0.20 × 0.15 mm
*Z* = 4	

### Data collection

**Table:**

Bruker SMART APEXII area-detector diffractometer	3169 independent reflections
Radiation source: fine-focus sealed tube	2133 reflections with *I* > 2σ(*I*)
Graphite monochromator	*R*_int_ = 0.033
ω and φ scans	θ_max_ = 28.3°, θ_min_ = 2.3°
Absorption correction: multi-scan (*SADABS*; Bruker, 2008)	*h* = −12→12
*T*_min_ = 0.667, *T*_max_ = 0.746	*k* = −14→18
12259 measured reflections	*l* = −14→13

### Refinement

**Table:**

Refinement on *F*^2^	Primary atom site location: structure-invariant direct methods
Least-squares matrix: full	Secondary atom site location: difference Fourier map
*R*[*F*^2^ > 2σ(*F*^2^)] = 0.053	Hydrogen site location: inferred from neighbouring sites
*wR*(*F*^2^) = 0.162	H atoms treated by a mixture of independent and constrained refinement
*S* = 1.03	*w* = 1/[σ^2^(*F*_o_^2^) + (0.0708*P*)^2^ + 0.303*P*] where *P* = (*F*_o_^2^ + 2*F*_c_^2^)/3
3169 reflections	(Δ/σ)_max_ < 0.001
158 parameters	Δρ_max_ = 0.26 e Å^−^^3^
0 restraints	Δρ_min_ = −0.21 e Å^−^^3^

### Special details

**Table:**

Geometry. Bond distances, angles etc. have been calculated using the rounded fractional coordinates. All su's are estimated from the variances of the (full) variance-covariance matrix. The cell esds are taken into account in the estimation of distances, angles and torsion angles
Refinement. Refinement of *F*^2^ against ALL reflections. The weighted *R*-factor *wR* and goodness of fit *S* are based on *F*^2^, conventional *R*-factors *R* are based on *F*, with *F* set to zero for negative *F*^2^. The threshold expression of *F*^2^ > σ(*F*^2^) is used only for calculating *R*-factors(gt) *etc*. and is not relevant to the choice of reflections for refinement. *R*-factors based on *F*^2^ are statistically about twice as large as those based on *F*, and *R*- factors based on ALL data will be even larger.

### Fractional atomic coordinates and isotropic or equivalent isotropic displacement parameters (Å^2^)

**Table:**

	*x*	*y*	*z*	*U*_iso_*/*U*_eq_	
O1	0.56542 (16)	0.15949 (11)	1.12382 (14)	0.0777 (5)	
O2	0.19690 (13)	0.02459 (8)	0.46195 (11)	0.0537 (4)	
O3	0.11891 (13)	0.03028 (9)	0.22128 (11)	0.0565 (4)	
N1	0.10656 (17)	0.13011 (11)	0.58487 (15)	0.0547 (5)	
C1	0.5636 (3)	0.09930 (17)	1.2298 (2)	0.0825 (8)	
C2	0.4475 (2)	0.14896 (12)	0.99327 (18)	0.0562 (6)	
C3	0.4759 (2)	0.17933 (13)	0.88187 (19)	0.0591 (6)	
C4	0.36687 (19)	0.17229 (13)	0.74810 (18)	0.0563 (6)	
C5	0.22273 (18)	0.13465 (11)	0.72040 (17)	0.0477 (5)	
C6	0.1945 (2)	0.10636 (13)	0.83313 (19)	0.0599 (6)	
C7	0.3056 (2)	0.11259 (13)	0.96875 (19)	0.0641 (7)	
C8	0.14159 (19)	0.12184 (12)	0.46695 (17)	0.0521 (5)	
C9	0.0083 (2)	0.14104 (13)	0.33069 (18)	0.0573 (6)	
C10	0.0541 (2)	0.12534 (12)	0.21047 (18)	0.0585 (6)	
C11	0.24203 (19)	0.01538 (14)	0.35085 (18)	0.0591 (6)	
C12	0.3020 (3)	−0.08501 (18)	0.3532 (2)	0.0865 (9)	
C13	−0.0748 (2)	0.13359 (16)	0.0683 (2)	0.0753 (7)	
H1	0.028 (2)	0.0955 (14)	0.576 (2)	0.065 (6)*	
H1A	0.65040	0.11310	1.31480	0.1240*	
H1B	0.56700	0.03290	1.20440	0.1240*	
H1C	0.47210	0.11050	1.24280	0.1240*	
H3	0.57060	0.20510	0.89760	0.0710*	
H4	0.38920	0.19290	0.67460	0.0680*	
H6	0.09880	0.08260	0.81800	0.0720*	
H7	0.28420	0.09220	1.04290	0.0770*	
H8	0.22250	0.16810	0.47710	0.0630*	
H9A	−0.02650	0.20710	0.32900	0.0690*	
H9B	−0.07490	0.09790	0.32100	0.0690*	
H10	0.13190	0.17330	0.21760	0.0700*	
H11	0.32240	0.06250	0.36270	0.0710*	
H12A	0.33440	−0.09200	0.27890	0.1300*	
H12B	0.22330	−0.13120	0.34140	0.1300*	
H12C	0.38680	−0.09610	0.44080	0.1300*	
H13A	−0.03690	0.12280	−0.00170	0.1130*	
H13B	−0.11850	0.19710	0.05700	0.1130*	
H13C	−0.15100	0.08610	0.05900	0.1130*	

### Atomic displacement parameters (Å^2^)

**Table:**

	*U*^11^	*U*^22^	*U*^33^	*U*^12^	*U*^13^	*U*^23^
O1	0.0820 (9)	0.0918 (10)	0.0602 (8)	−0.0148 (8)	0.0302 (7)	0.0000 (7)
O2	0.0582 (6)	0.0538 (7)	0.0540 (7)	0.0064 (5)	0.0281 (5)	0.0009 (5)
O3	0.0587 (7)	0.0601 (7)	0.0520 (7)	−0.0045 (5)	0.0243 (6)	−0.0050 (5)
N1	0.0539 (8)	0.0563 (9)	0.0579 (9)	−0.0016 (6)	0.0270 (7)	−0.0055 (6)
C1	0.1004 (16)	0.0807 (14)	0.0697 (13)	0.0130 (12)	0.0384 (12)	0.0102 (11)
C2	0.0630 (10)	0.0509 (9)	0.0562 (10)	−0.0005 (8)	0.0262 (8)	−0.0032 (7)
C3	0.0561 (9)	0.0619 (11)	0.0642 (11)	−0.0034 (8)	0.0297 (9)	0.0005 (8)
C4	0.0596 (10)	0.0594 (10)	0.0575 (10)	0.0003 (8)	0.0317 (8)	0.0031 (8)
C5	0.0566 (9)	0.0365 (7)	0.0551 (9)	0.0039 (6)	0.0281 (8)	−0.0046 (6)
C6	0.0675 (10)	0.0547 (10)	0.0676 (11)	−0.0132 (8)	0.0381 (9)	−0.0071 (8)
C7	0.0876 (13)	0.0588 (11)	0.0595 (11)	−0.0108 (9)	0.0439 (10)	−0.0019 (8)
C8	0.0576 (9)	0.0466 (9)	0.0546 (10)	−0.0039 (7)	0.0258 (8)	−0.0028 (7)
C9	0.0642 (10)	0.0461 (9)	0.0591 (10)	0.0038 (7)	0.0228 (8)	0.0013 (7)
C10	0.0661 (10)	0.0501 (10)	0.0569 (10)	−0.0124 (8)	0.0231 (8)	0.0030 (7)
C11	0.0503 (9)	0.0752 (12)	0.0559 (10)	−0.0014 (8)	0.0259 (8)	−0.0070 (8)
C12	0.0845 (14)	0.1010 (17)	0.0764 (14)	0.0342 (12)	0.0356 (12)	−0.0071 (12)
C13	0.0867 (14)	0.0712 (13)	0.0582 (11)	−0.0038 (11)	0.0200 (10)	0.0090 (9)

### Geometric parameters (Å, º)

**Table:**

O1—C1	1.399 (3)	C1—H1A	0.9600
O1—C2	1.385 (2)	C1—H1B	0.9600
O2—C8	1.455 (2)	C1—H1C	0.9600
O2—C11	1.413 (2)	C3—H3	0.9300
O3—C10	1.439 (2)	C4—H4	0.9300
O3—C11	1.407 (2)	C6—H6	0.9300
N1—C5	1.407 (2)	C7—H7	0.9300
N1—C8	1.421 (2)	C8—H8	0.9800
N1—H1	0.87 (2)	C9—H9A	0.9700
C2—C7	1.379 (3)	C9—H9B	0.9700
C2—C3	1.377 (3)	C10—H10	0.9800
C3—C4	1.371 (3)	C11—H11	0.9800
C4—C5	1.398 (3)	C12—H12A	0.9600
C5—C6	1.380 (3)	C12—H12B	0.9600
C6—C7	1.391 (3)	C12—H12C	0.9600
C8—C9	1.504 (2)	C13—H13A	0.9600
C9—C10	1.520 (3)	C13—H13B	0.9600
C10—C13	1.506 (3)	C13—H13C	0.9600
C11—C12	1.499 (3)		
			
C1—O1—C2	117.08 (17)	C4—C3—H3	119.00
C8—O2—C11	110.86 (13)	C3—C4—H4	119.00
C10—O3—C11	112.07 (13)	C5—C4—H4	119.00
C5—N1—C8	120.95 (16)	C5—C6—H6	119.00
C8—N1—H1	111.9 (13)	C7—C6—H6	119.00
C5—N1—H1	115.4 (13)	C2—C7—H7	120.00
O1—C2—C7	124.74 (17)	C6—C7—H7	120.00
O1—C2—C3	116.39 (18)	O2—C8—H8	109.00
C3—C2—C7	118.86 (17)	N1—C8—H8	109.00
C2—C3—C4	121.14 (19)	C9—C8—H8	109.00
C3—C4—C5	121.08 (17)	C8—C9—H9A	110.00
C4—C5—C6	117.26 (16)	C8—C9—H9B	110.00
N1—C5—C6	120.25 (17)	C10—C9—H9A	110.00
N1—C5—C4	122.40 (16)	C10—C9—H9B	110.00
C5—C6—C7	121.66 (19)	H9A—C9—H9B	108.00
C2—C7—C6	119.98 (18)	O3—C10—H10	108.00
N1—C8—C9	113.75 (16)	C9—C10—H10	108.00
O2—C8—N1	109.21 (13)	C13—C10—H10	108.00
O2—C8—C9	108.07 (13)	O2—C11—H11	109.00
C8—C9—C10	110.04 (16)	O3—C11—H11	109.00
O3—C10—C13	107.55 (14)	C12—C11—H11	109.00
C9—C10—C13	114.46 (17)	C11—C12—H12A	109.00
O3—C10—C9	109.30 (14)	C11—C12—H12B	110.00
O3—C11—C12	108.48 (15)	C11—C12—H12C	109.00
O2—C11—O3	111.45 (15)	H12A—C12—H12B	109.00
O2—C11—C12	108.56 (16)	H12A—C12—H12C	109.00
O1—C1—H1A	109.00	H12B—C12—H12C	109.00
O1—C1—H1B	109.00	C10—C13—H13A	109.00
O1—C1—H1C	109.00	C10—C13—H13B	109.00
H1A—C1—H1B	109.00	C10—C13—H13C	109.00
H1A—C1—H1C	110.00	H13A—C13—H13B	109.00
H1B—C1—H1C	109.00	H13A—C13—H13C	109.00
C2—C3—H3	119.00	H13B—C13—H13C	110.00
			
C1—O1—C2—C3	−156.71 (19)	O1—C2—C3—C4	−179.91 (17)
C1—O1—C2—C7	24.7 (3)	O1—C2—C7—C6	179.13 (17)
C11—O2—C8—N1	−176.31 (14)	C3—C2—C7—C6	0.5 (3)
C11—O2—C8—C9	59.47 (19)	C7—C2—C3—C4	−1.2 (3)
C8—O2—C11—O3	−62.20 (18)	C2—C3—C4—C5	0.5 (3)
C8—O2—C11—C12	178.39 (16)	C3—C4—C5—N1	177.38 (17)
C11—O3—C10—C9	−55.69 (19)	C3—C4—C5—C6	0.9 (3)
C11—O3—C10—C13	179.51 (15)	N1—C5—C6—C7	−178.12 (16)
C10—O3—C11—O2	60.39 (19)	C4—C5—C6—C7	−1.6 (3)
C10—O3—C11—C12	179.85 (17)	C5—C6—C7—C2	0.9 (3)
C8—N1—C5—C4	28.2 (2)	O2—C8—C9—C10	−55.53 (18)
C8—N1—C5—C6	−155.40 (16)	N1—C8—C9—C10	−176.99 (14)
C5—N1—C8—O2	72.63 (19)	C8—C9—C10—O3	53.91 (19)
C5—N1—C8—C9	−166.56 (15)	C8—C9—C10—C13	174.58 (15)

### Hydrogen-bond geometry (Å, º)

Cg1 is the centroid of ring C2-C7 ring.

**Table:**

*D*—H···*A*	*D*—H	H···*A*	*D*···*A*	*D*—H···*A*
C6—H6···O3^i^	0.93	2.51	3.407 (2)	162
C1—H1*B*···*Cg*1^ii^	0.96	2.77	3.700 (3)	163

Symmetry codes: (i) −*x*, −*y*, −*z*+1; (ii) −*x*+1, −*y*, −*z*+2.
